# Feasibility of a Microarray-Based Point-of-Care *CYP2C19* Genotyping Test for Predicting Clopidogrel On-Treatment Platelet Reactivity

**DOI:** 10.1155/2013/154073

**Published:** 2013-03-28

**Authors:** Hyojin Chae, Myungshin Kim, Yoon-Seok Koh, Byung-Hee Hwang, Min-Kyu Kang, Yonggoo Kim, Hae-il Park, Kiyuk Chang

**Affiliations:** ^1^Department of Laboratory Medicine, Bucheon St. Mary's Hospital, 2 Sosa-dong, Wonmi-gu, Gyeonggi-do, Bucheon-si 420-717, Republic of Korea; ^2^Catholic Laboratory Development and Evaluation Center, College of Medicine, The Catholic University of Korea, Seoul 137-701, Republic of Korea; ^3^Cardiovascular Center and Cardiology Division, Uijeongbu St. Mary's Hospital, Uijeongbu 480-717, Republic of Korea; ^4^Cardiovascular Center and Cardiology Division, Seoul St. Mary's Hospital, 505 Banpo-dong, Seocho-gu, Seoul 137-701, Republic of Korea

## Abstract

Clopidogrel is a prodrug which is converted into active metabolite by cytochrome P450 isoenzyme, *CYP2C19*. Numerous polymorphisms of *CYP2C19* are reported, and a strong link exists between loss-of-function (LOF) or gain-of-function polymorphisms, clopidogrel metabolism, and clinical outcome. Hence, a fully automated point-of-care CYP2C19 genotyping assay is more likely to bring personalized antiplatelet therapy into real practice. We assessed the feasibility of the Verigene 2C19/CBS Nucleic Acid Test, a fully automated microarray-based assay, compared to bidirectional sequencing, and performed VerifyNow P2Y12 assay to evaluate the effect of *CYP2C19* polymorphisms on on-treatment platelet reactivity in 57 Korean patients treated with clopidogrel after percutaneous coronary intervention. The Verigene 2C19/CBS assay identified ∗2, ∗3, and ∗17 polymorphisms with 100% concordance to bidirectional sequencing in 180 minutes with little hands-on time. Patients were classified into 4 groups: extensive (∗1/∗1; *n* = 12, 21.1%), intermediate (∗1/∗2, ∗1/∗3; *n* = 33, 57.9%), poor (∗2/∗2, ∗2/∗3, and ∗3/∗3; *n* = 11, 19.3%), and ultrarapid metabolizers (∗1/∗17; *n* = 1, 1.8%). The prevalence of the *CYP2C19*  ∗2, ∗3, and ∗17 alleles was 36.0%, 12.3%, and 0.9%. Platelet reactivity showed gene dose response according to the number of *CYP2C19* LOF allele. In conclusion, the Verigene 2C19/CBS assay gave accurate *CYP2C19* genotype results which were in well match with the differing on-treatment platelet reactivity.

## 1. Introduction

Clopidogrel is a thienopyridine prodrug, whose active liver metabolite covalently binds cysteine residues of the platelet surface P2Y12 receptor, irreversibly blocking the receptor, leading to inhibition of platelet aggregation in response to ADP and also to other agents through the inhibitory effect on released ADP.

The conversion of clopidogrel to its active metabolite relies on the CYP2C19 enzyme, a member of the hepatic cytochrome P450 family. Numerous polymorphisms in *CYP2C19* have been identified and individuals can be classified as the phenotype of extensive metabolizers (EMs), intermediate metabolizers (IMs), poor metabolizers (PMs), and ultrarapid metabolizers (UMs) according to the polymorphism of *CYP2C19*. Among the PM phenotypes, *CYP2C19*  *2 and *3 polymorphisms are the most frequent and both alleles confer loss-of-function (LOF) leading to a complete loss of the enzyme activity. Indeed, in patients who carry the *CYP2C19*  *2 or *3 allele, the conversion of clopidogrel to its active metabolite is reduced, resulting in decreased response of platelets to clopidogrel treatment and worse cardiovascular outcome.

Clopidogrel on top of aspirin has revolutionized the treatment of patients with acute coronary syndrome and undergoing percutaneous coronary intervention (PCI) [1, 2]. However, interindividual variation of platelet inhibition by clopidogrel has been noted, and from 5% to 11% of patients on clopidogrel treatment experience acute or subacute thrombosis after a coronary event or implantation of a coronary stent [1]. Accordingly, a black-box warning was added to the clopidogrel package insert indicating a significant clinical link between *CYP2C19 *LOF genotypes (*2 and *3) and poor metabolism of clopidogrel [3]. Therefore, a *CYP2C19* genotyping assay with a rapid sample-to-result time could be beneficial in the appropriate dosing of clopidogrel based on the genotype of the patient and/or permitting change to other antiplatelet agents in a timely manner.

The aim of this study was to assess the performance of the Verigene 2C19/CBS Nucleic Acid Test (Nanosphere, Northbrook, IL, USA), a fully automated microarray-based assay that identifies 12 allelic variants of *CYP2C19* (*1–*10, *13, and *17) in a rapid turnaround time of approximately 3 hours [3], and to study the influence of the *CYP2C19* allelic variants derived from the Verigene test on on-treatment platelet reactivity as assessed by VerifyNow P2Y12 assay (Accumetrics, San Diego, CA, USA) in Korean patients treated with clopidogrel after PCI with the use of drug-eluting stents (DESs).

## 2. Materials and Methods

### 2.1. Patients

Peripheral blood samples were collected from 57 consecutive patients subjected to PCI. All patients were preliminarily treated with 100 mg/day of aspirin followed by coadministration of clopidogrel (loading dose, 600 mg; maintenance dose, 75 mg/day). Exclusion criteria were platelet count outside from 100 to 450 × 10^9^/L range; hematocrit <25% or hemoglobin <8 g/dL; and chronic renal failure requiring dialysis. The study was conducted in accordance with the Declaration of Helsinki ethical guidelines and was approved by the Institutional Review Board at Catholic Medical Center.

### 2.2. Platelet Function Testing with VerifyNow P2Y12 Assay

The blood was drawn from the antecubital vein at 48 h after clopidogrel loading dose into a 3.2% sodium citrate tube for the VerifyNow P2Y12 assay. The time interval between blood sampling and VerifyNow P2Y12 testing did not exceed 2 hours. Platelet reactivity was assessed by the VerifyNow P2Y12 assay, and the VerifyNow P2Y12 assay was performed as previously described [4]. With this assay, higher P2Y12 reaction units (PRU) reflect greater ADP-mediated platelet reactivity. Cut-off value for high on-treatment residual ADP-inducible platelet reactivity (HRPR) was PRU > 235 for the VerifyNow P2Y12 assay according to the published consensus statement [5].

### 2.3. Genotyping with Verigene 2C19/CBS Nucleic Acid Test

The blood was drawn from the antecubital vein into an EDTA tube for genotyping. For the Verigene 2C19/CBS Nucleic Acid Testing, the EDTA-anticoagulated whole blood (EDTA-WB) samples could be stored at 2–8°C for up to 10 days before processing. Briefly, a single-use extraction tray containing all necessary reagents to lyse, extract, and purify DNA from WB was loaded into the Verigene Processor SP (Nanosphere). 1.0 mL of EDTA-WB was transferred to the sample well in the extraction tray. A single-use test cartridge containing the slide array and hybridization reagents was loaded into the Verigene Processor SP, and the assay was started. On completion of the assay, the test cartridge was removed from the processor, and the hybridization slide was inserted into the Verigene Reader.

### 2.4. Direct Sequencing

To evaluate the accuracy of the genotype results obtained with the Verigene 2C19/CBS Nucleic Acid Test we performed Sanger-based direct sequencing method in parallel. Briefly, the Genomic DNA was isolated from the peripheral leukocytes using the QIAmp DNA Mini Kit (Qiagen, Hamburg, Germany). PCR was carried out using previously published primer sets for *2 and *3 [6] and a newly designed primer set for *17. The PCR amplicons were sequenced using the Big Dye terminator v3.1 cycle sequencing kit (Applied Biosystems, Foster City, CA, USA) on an ABI PRISM 3100 Genetic Analyzer (Applied Biosystems). The chromatograms were analyzed with the Sequencher software version 4.9 (Gene Codes).

### 2.5. Statistical Analysis

PRU values of VerifyNow P2Y12 assay were compared between the metabolizer statuses by one-way analysis of variance (ANOVA). MedCalc version 12.1.4 (Mariakerke, Belgium) was used for all statistical analyses; *P* < 0.05 was considered statistically significant.

## 3. Results

Clinical and laboratory characteristics of the total population are presented in Table 1. The average age was 67.0 years and about 65% of patients were men. According to the defined cut-off value, the frequency of clopidogrel resistance was 42.1%. Baseline demographics, clinical presentation, and treatment were well balanced between the *CYP2C19* LOF carrier/and noncarrier groups (*P* > 0.05).

### 3.1. Verigene 2C19/CBS Nucleic Acid Test for Identifying *CYP2C19* Polymorphisms

An initial result was obtained for 53 of 57 specimens (93.0%) using the Verigene 2C19/CBS Nucleic Acid Test. On retesting, all 4 samples gave a definitive result. The total time-to-result was approximately 3 hours with about 15 minutes of hands-on time. The comparison of polymorphism results between bidirectional sequencing and the Verigene 2C19/CBS Nucleic Acid Test revealed 100% concordance rate for all 57 specimens that were analyzed.

### 3.2. Genotype Frequencies and Classification of Metabolizer Statuses

Distributions of the *CYP2C19* alleles, genotypes, and the predicted phenotypes in our study population are given in Table 2. The frequency of the LOF genotype was high (77%). The prevalence of the gain-of-function variant, *CYP2C19*  *17 allele, was low (1%), and the prevalence of the *CYP2C19*  *2 and *3 alleles were 36% and 12%, respectively. Of the 57 patients included in the study, 12 (21%) were classified as extensive (EM), 1 (2%) as ultrarapid (UM), 33 (58%) as intermediate (IM), and 11 (19%) as poor (PM) metabolizers.

### 3.3. Influence of Metabolizer Statuses on Platelet Reactivity

**Figure 1:**
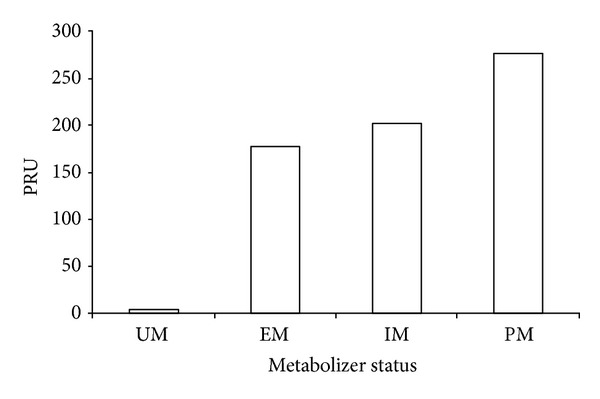
Result from the VerifyNow P2Y12 assay according to CYP2C19 metabolizer statuses. Data are shown as mean. The denotations are PRY: P2Y12 reaction units; UM: ultrarapid; EM: extensive; IM: intermediate; and PM: poor metabolizer.

Platelet reactivity measured by the VerifyNow P2Y12 assay differed significantly according to metabolizer statuses when tested by analysis of variance (Figure 1). Higher on-clopidogrel platelet reactivity was observed as the number of *CYP2C19* LOF allele increased (UM 4.0; EM, 177.7; IM, 201.7; and PM 277.0). Student-Newman-Keuls test for pairwise comparison revealed a significant difference between UM and IM/PM using the VerifyNow P2Y12 assay.

## 4. Discussion

In this study, we found that the test reliability of the Verigene 2C19/CBS Nucleic Acid Test for the identification of *CYP2C19* polymorphisms was 100% accurate as compared with the bidirectional sequencing method. In addition, this Verigene test offered rapid detection time and enabled point-of-care diagnosis without conventional DNA extraction, PCR steps, and sequencing. Genotyping assays for *CYP2C19* can be performed with numerous molecular methods, such as real-time PCR, allele-specific PCR, PCR-RFLP, pyrosequencing, and bidirectional sequencing. Also a growing number of commercial analytical platforms are available, and these include the INFINITI (AutoGenomics, Inc., Vista, Carlsbad, CA, USA) [7], the Verigene, eSensor XT-8 (Genmark, Carlsbad, CA, USA) [8], Spartan RX (Spartan Biosciences, Ottawa, ON, Canada) [9], Invader (Hologic, Bedford, MA, USA) [10], and Luminex assays (Luminex, Austin, TX, USA) [8]. Although the comparison of different genotyping methods is beyond the scope of this paper, the Verigene assay utilizes signal amplification and not target amplification, and therefore it has a unique advantage of operating in a PCR-free environment. Also Verigene has the shortest TATs among commercial platforms that are capable of genotyping a certain number of polymorphisms and also the least complex to operate. And therefore the assay can be readily implemented in clinical laboratories without extensive experience in molecular techniques. The limitation of the Verigene assay is the no call rate of 7–10%. The rate of no call at first attempt was 7.3% in our study; however, in our experience, the no call errors were resolved with repeat testing.

The inhibitory effect of clopidogrel on platelet function shows marked interindividual variability. The prevalence of clopidogrel nonresponsiveness has been reported to be from 4% to 30% in patients with coronary artery disease [11]. Several factors including underdosing, inappropriate dosing, variable absorption of the prodrug, variable clearance of the active metabolite, potential drug-drug interactions, P2Y12 receptor variability, and genetic polymorphisms of cytochrome P450 isoenzymes are possible mechanisms of clopidogrel resistance [11]. In this respect, *CYP2C19* polymorphisms specifically addresses the issues associated with dosing, since *in vivo* transformation of the prodrug to its active metabolite is dependent on the hepatic cytochrome P450 isoenzyme CYP2C19. Today, at least 25 single nucleotide polymorphisms (SNPs) in the gene coding for CYP2C19 have been described. Of these, *CYP2C19*  *2, *3 are the most frequent LOF polymorphisms and thus the main genetic determinants of clopidogrel response variability. In this study, we also observed that *CYP2C19* LOF alleles were significantly associated with reduced antiplatelet efficacy of clopidogrel.

There exists a marked interracial difference in the frequency of the *CYP2C19* polymorphisms. Asians have a higher prevalence of *CYP2C19* LOF alleles and PM phenotypes (from 13% to 23% in Asians and from 1% to 6% in Caucasians) [12, 13]. In this study, the prevalence of *CYP2C19* PM, *2, and *3 alleles were 19.3%, 36.0%, and 12.3%, respectively. These results are in agreement with the observed allele frequencies of 28.6% and 7.4% for *CYP2C19*  *2 and *3 alleles in a previous report of 200 Korean individuals [14]. The carriage prevalence of the *CYP2C19* LOF variant is 77.2% in this study, which is also in line with the reported prevalence of 55% to 70% among Asians [12].

Multiple tests are available for the monitoring of antiplatelet therapy, especially aspirin and clopidogrel. Light transmission aggregometry (LTA) is the gold standard for assessing the platelet response to ADP [4], but this method is laborious and weakly standardized. In the present study, the VerifyNow P2Y12 assay, a method that shows the strongest correlation with the LTA among whole blood-based methods, was used to assess clopidogrel-mediated platelet inhibition. Platelet reactivity measured by the VerifyNow P2Y12 assay significantly differed according to metabolizer statuses when tested by analysis of variance in our population. Higher on-clopidogrel platelet reactivity was observed as the number of *CYP2C19* LOF allele increased (UM 4.0; EM, 177.7; IM, 201.7; and PM 277.0), but post hoc analysis did not reach statistical significance for linear trend, probably as a result of the small number of patients. Interestingly, a rare but well-recognized gain of function allelic variant *CYP2C19*  *17 was identified in our study. Student-Newman-Keuls test for pairwise comparison revealed a significant difference between UM and both IM/PM platelets reactivity using the VerifyNow P2Y12 assay.

The Verigene 2C19/CBS Nucleic Acid Test is a fully automated microarray test that utilizes gold nanoparticle-conjugated oligonucleotide probes to detect nucleic acids captured by array probes, and this method eliminates the need for target amplification, namely, a PCR step, before array hybridization [3, 15]. Therefore the assay is less prone to errors introduced during the conventional nucleic acid extraction and target amplification processes and has a rapid turn-around-time. The initial call rate of this study was 93.0% which is similar to the reported initial call rate of 93.5% in a previous study [3], and the assay identified heterozygous and homozygous *2, *3, and *17 polymorphisms with 100% concordance to bidirectional sequence analysis in 57 patient samples. Most importantly, the total time to result was approximately 3 hours with less than 15 minutes of hands-on time.

There are a number of limitations of this study. Our cohort consisted of only 57 patient samples and the small sample size related to lack of statistical power. Also another limitation of our study was the use of PRU values as measured by VerifyNow P2Y12 assay as a measure of clinical efficacy and did not include clinical endpoints. However, the analytical validation of Verigene assay as well as clinical validation using PRU values as surrogate endpoints to clinical efficacy serves a crucial role in providing the link of the point-of-care microarray CYP2C19 genotyping assay towards pharmacogenetic dosing of clopidogrel in real clinical practice.

## 5. Conclusion

In conclusion, the concordance rate of the Verigene 2C19/CBS Nucleic Acid Test with Sanger's sequencing method was 100% in this study of Korean patients treated with clopidogrel after coronary stenting. In addition, the genetic test results of the *CYP2C19* polymorphisms highly predicted the on-treatment platelet reactivity as assessed by VerifyNow P2Y12 assay. The Verigene test offered several advantages for the detection of *CYP2C19* polymorphisms such as easiness of use, rapid detection time, and a lower test error rate and test failure rate. We anticipate that the rapid point-of-care *CYP2C19* genetic test will clarify the clinical utility of clopidogrel pharmacogenetic tests in patients treated with clopidogrel.

## Figures and Tables

**Table 1:** Baseline characteristics of the study population.

	Overall	LOF	No LOF	*P* value
	*N* = 56	*N* = 44	*N* = 12	
Age	67.0	67.0	66.0	0.7
Male gender	36 (64.3)	29 (65.9)	7 (58.3)	0.7
Smoking	10 (17.9)	7 (15.9)	3 (25.0)	0.4
Hypertension	33 (58.9)	25 (56.8)	8 (66.7)	0.7
Diabetes mellitus	15 (26.8)	11 (25.0)	4 (33.3)	0.7
Dyslipidemia	12 (21.4)	9 (20.5)	3 (25.0)	0.7
Prior MI	3 (5.4)	1 (2.3)	2 (16.7)	0.1
Prior PCI	11 (19.6)	7 (15.9)	4 (33.3)	0.2
BMI	24.8	26.1	24.3	0.2
Statin	15 (26.8)	13 (29.5)	2 (16.7)	0.5
*β*-blocker	9 (16.1)	5 (11.4)	4 (33.3)	0.09
ACE inhibitor	4 (7.1)	3 (6.8)	1 (8.3)	1
CCBs	11 (19.6)	8 (18.2)	3 (25.0)	0.7
PPIs	2 (3.6)	1 (2.3)	1 (8.3)	0.4
Platelet count	215.5	221.0	215.5	1
C-reactive protein	0.1	0.1	0.1	0.2

The denotations are LOF: loss-of-function; MI: myocardial infarction; PCI: percutaneous coronary intervention; BMI: body mass index; ACE: angiotensin converting enzyme inhibitor; CCB: calcium channel blocker; and PPI: proton pump inhibitor.

**Table 2:** Distributions of the *CYP2C19* alleles, genotypes, and the predicted phenotypes.

Allele	Frequency, *n* (%)	Genotype	Frequency, *n* (%)	Phenotype	Metabolizer status
*1	58 (51)	*17/wt	1 (2)	Rapid heterozygous	UM
*2	41 (36)	wt/wt	12 (21)	Extensive	EM
*3	14 (12)	*2/wt	24 (42)	Intermediate	IM
*17	1 (1)	*3/wt	9 (16)	Intermediate	IM
		*2/*2	6 (10)	Poor	PM
		*2/*3	5 (9)	Poor	PM

The denotations are wt: wild type; UM: ultrarapid metabolizer; EM: extensive metabolizer; IM: intermediate metabolizer; and PM: poor metabolizer.
